# An integrated overview of spatiotemporal organization and regulation in mitosis in terms of the proteins in the functional supercomplexes

**DOI:** 10.3389/fmicb.2014.00573

**Published:** 2014-10-29

**Authors:** Yueyuan Zheng, Junjie Guo, Xu Li, Yubin Xie, Mingming Hou, Xuyang Fu, Shengkun Dai, Rucheng Diao, Yanyan Miao, Jian Ren

**Affiliations:** ^1^Cancer Center, School of Life Sciences, School of Advanced Computing, Cooperative Innovation Center for High Performance Computing, Sun Yat-sen UniversityGuangzhou, China; ^2^Orthopaedic Department of Anhui Medical University Affiliated Provincial HospitalHefei, China

**Keywords:** super-complex structures, cell division/mitosis, protein components, centrosome, kinetochore, midbody

## Abstract

Eukaryotic cells may divide via the critical cellular process of cell division/mitosis, resulting in two daughter cells with the same genetic information. A large number of dedicated proteins are involved in this process and spatiotemporally assembled into three distinct super-complex structures/organelles, including the centrosome/spindle pole body, kinetochore/centromere and cleavage furrow/midbody/bud neck, so as to precisely modulate the cell division/mitosis events of chromosome alignment, chromosome segregation and cytokinesis in an orderly fashion. In recent years, many efforts have been made to identify the protein components and architecture of these subcellular organelles, aiming to uncover the organelle assembly pathways, determine the molecular mechanisms underlying the organelle functions, and thereby provide new therapeutic strategies for a variety of diseases. However, the organelles are highly dynamic structures, making it difficult to identify the entire components. Here, we review the current knowledge of the identified protein components governing the organization and functioning of organelles, especially in human and yeast cells, and discuss the multi-localized protein components mediating the communication between organelles during cell division.

## Introduction

Cell division/mitosis is a precisely modulated process of chromosome segregation and nuclear division in which one eukaryotic cell divides into two daughter cells with identical chromosomes in order to produce more cells for growth and replace any damaged, dying or senescent cells (Sancar et al., 2004). Mitosis is always accompanied by a separation of the cell cytoplasm, known as cytokinesis, in which the daughter cells become completely separated (Wheatley et al., 2001; Straight et al., 2003). Mitosis (nuclear division) and cytokinesis (cytoplasmic division), which define the mitotic (M) phase, are the most crucial and fundamental activities of the eukaryotic cell cycle. Before entering the M phase of the cell cycle, the cell undergoes a period of growth and maturation during the interphase, duplicating genetic materials and organelles for the performance of cell division (Heun et al., 2001). The interphase and M phase of the cell cycle are complex and highly regulated by numerous proteins which are spatially and temporally organized as protein super-complexes. The super-complexes carry out chromosome replication and alignment, sister chromatid separation and cytoplasm division (Straight et al., 2003).

Chromosome must be precisely replicated once per cell cycle to maintain genome integrity. Eukaryotic cells may use multiple proteins, many of which are also involved in super-complex formation to regulate chromosome alignment, separation and cytoplasm division, to control the origins of chromosome replication. During the interphase, the origin recognition complex (ORC), a six-subunit complex comprised of ORC1-6, binds to chromosomes at the replication origin sites and acts as a central component for eukaryotic chromosome replication initiation (Bell and Dutta, 2002). As the initiation of replication is a central event in cell cycle, the identification of replication origin sites and its binding proteins is essential to the understanding of DNA replication. Benefit from recent genome-wide approaches, a huge number of replication origins and ORC proteins were identified. Also, several specialized databases, such as DeOri (Gao et al., 2012), have been developed to assist the comprehensive study on eukaryotic DNA replication. Over the years, new roles for many ORC proteins were revealed in cells. Unlike their regular function that controls the initiation of DNA replication, a fair amount of ORC proteins also binds to other cell cycle-related organelles, including centrosome, kinetochore and midbody. Evidences have shown that ORC1 and ORC2 can regulate centrosome duplication and a depletion of them resulted in abnormal centrosome copy number (Prasanth et al., 2004; Hemerly et al., 2009). Coincidentally, researches also demonstrated that ORC6 and ORC2 can localize to kinetochore. The absence of ORC proteins may lead to kinetochore dysfunction (Shimada and Gasser, 2007). Furthermore, in anaphase, the ORC6 may target to the midbody in controlling of chromosome segregation (Prasanth et al., 2002). In the process of chromosome replication, the enzymes that catalyze DNA duplication are unable to reach the very end of the chromosome. Chromosome has a special DNA structure named telomere at the end. Thus, in the course of each replication, the length of the telomeres is shortened (Von Zglinicki, 2002). Once the telomeres shrink to a critical minimum size, the cells no longer divide and ultimately become senescent or die (Hahn et al., 1999; Henson et al., 2002). However, telomerase, a unique protein-RNA complex that is activated in certain cells (Rudolph et al., 1999; Hanahan and Weinberg, 2000), such as yeast cells, stem cells, reproductive cells and cancer cells, is responsible for elongating telomeres (Herbert et al., 1999; Dunham et al., 2000). It thus prevents the chromosome degradation, maintains the stability of the genome and assists cells to escape the fate of being unable to continue division (Hoeijmakers, 2001).

In the M phase of the cell cycle, multiple proteins assemble in the three distinct regions of the centrosome/spindle pole body, kinetochore/centromere and cleavage furrow/midbody/bud neck, directing the process of cell division. The centrosomes in animal cells, spindle pole bodies (SPB) in budding yeast and related/homologous structures in other organisms have been characterized as the microtubule organizing centers (MTOCs) (Veith et al., 2005), which participate in the organization and orientation of the mitotic spindle apparatus, and thus direct the chromosome alignment and sister chromatids segregation during cell division. In addition, the kinetochore, a specialized protein complex which is dynamically assembled around the centromere of chromosomes (Ditchfield et al., 2003), acts as the “handle” of the chromosome and specifies the attachments between the chromosomes and spindle to ensure accurate chromosome segregation (Hauf et al., 2003). Dysfunction of the centrosome/spindle pole body and kinetochore/centromere is catastrophic for cells and contributes to aberrant division and chromosome instability (Fodde et al., 2001), both of which are hallmarks of cancer cells (Schuyler et al., 2012). The chromosome separation in animal cells is always accompanied by cytokinesis, which begins with ingression of the cleavage furrow mediated by the actomyosin ring (Somers and Saint, 2003), followed by the formation dense structure of the midbody (Gromley et al., 2005) which is also known as the phragmoplast in plants (Van Damme et al., 2004) and the bud neck in budding yeast (Vallen et al., 2000; Caviston et al., 2003). Numerous proteins are recruited to the midbody and form a super-complex which mediates the midbody abscission in order to perform cytokinesis, with complete separation of the two daughter cells (Adams et al., 2001; Wheatley et al., 2001; Mollinari et al., 2002).

Although the importance of organelles to cell biology has been repeatedly demonstrated by multiple reports over the past decades, many aspects of their function, structure and composition are still largely unknown. In this regard, comprehensive identification of the protein components of the super-complex structures will be one of the keys to understanding the mechanisms of chromosome segregation and cytokinesis, and may provide important clues for the discovery and validation of new therapeutic targets. Recently, many protein components have been identified, but according to a combination of proteomic analysis, biochemical studies and genetic screening, there still remain a large number of proteins that are predicted to be associated with these organelles (Table 1). In this review, we will present a general overview of the identified components of the super-complexes involved in the mitosis and cytokinesis with the aim of integrating the relevant information of organelles and thus broadening the knowledge of cell division. Remarkably, the process of the cell division is highly conserved in eukaryotic cells, we therefore briefly review the two commonly studied systems, human and yeast cells.

**Table 1:** **The number of proteins located in centrosome, kinetochore, midbody with experimental verification and predicted in 7 different species from MiCroKiTS (Updated June 27, 2014)**.

**Organism**	**Centrosome**		**Kinetochore**		**Midbody**	
	**Experimental verification**	**Predicted from orthologs**	**Experimental verification**	**Predicted from orthologs**	**Experimental verification**	**Predicted from orthologs**
*H. sapiens*	516	112	203	82	229	92
*M. musculus*	131	477	33	249	22	289
*X. laevis*	36	0	29	0	6	0
*C. elegans*	35	227	59	129	20	148
*D. melanogaster*	67	240	58	146	29	152
*S. cerevisiae*	89	107	102	56	133	50
*S. pombe*	48	145	91	66	38	108

## The centrosome

As a complex and dynamic organelle, the MTOC contributes to both microtubule organization and nucleation, which are important for chromosomes separation during mitosis (Brinkley, 1985; Luders and Stearns, 2007). Multiple proteins must be involved in manipulating MTOC functions, controlling its duplication and driving maturation. To further clarify the functional processes of the organization and regulation of the MTOC, the protein components must be identified. Recently, evidence from a combination of genetic and biochemical studies has revealed many important MTOC-associated proteins in a variety of species (Masuda et al., 2013). However, according to the MiCroKiTS database (Ren et al., 2010) (http://microkit.biocuckoo.org/), an integrated database of the midbody, centrosome and kinetochore most recently updated in June 27, 2014, a large number of proteins that are predicted to be located on the MTOC are still not well validated (Table 1). Confirmation of the functions of these predicted proteins has broad implications for the understanding of the MTOC.

The centrosome is the primary MTOC, which contains two orthogonally arranged centrioles and the surrounding pericentriolar material (PCM) (Nigg and Raff, 2009). The centriole, composed mainly of tublin, is a typically cylindrical organelle made up of nine triplets of microtubules in most animal eukaryotic cells (Kitagawa et al., 2011), although absent in most fungi and high plant cells (Bell and Dutta, 2002; Gao et al., 2012). In the G1 phase of the cell cycle, the paired centrioles, termed the mother and the daughter centrioles, are connected via interconnecting fibers. Morphologically distinct from the daughter centriole, the mother centriole has both distal and subdistal appendages that serve to anchor the centrioles to the plasma membrane (Bettencourt-Dias and Glover, 2009). Recently, several components of the centriole appendages have been described, such as the distal appendage proteins CEP164 and CEP89, as well as three novel components of CEP83, the Sodium channel and clathrin linker 1 (SCLT1) and the Fas-binding factor 1 (FBF1) (Tanos et al., 2013; Kloc et al., 2014). The subdistal appendage proteins include Outer dense fiber 2 (ODF2; also known as cenexin) (Chang et al., 2003), ninein (Graser et al., 2007), epsilon-tubulin (Chang et al., 2003), Centriolin (Gromley et al., 2003), and CC2D2A (Veleri et al., 2014). However, the molecular composition and the exact functions of the appendages remain largely unclear. The mechanisms underlying the assembly of the centriole are still poorly understood. In recent years, identification of the proteins that are responsible for centriole formation has advanced the understanding of the assembly mechanisms. In human cells, spindle assembly abnormal 6 (HsSAS-6), Polo-like kinase 4 (PLK4), SCL/TAL1 interrupting locus (STIL), centrosomal P4.1-associated protein (CPAP) (Brownlee and Rogers, 2013), located at the centriole, have been identified as the core components required for centriole assembly. Using proteomic and biochemical analysis as well as genetic screening, a list of the proteins associated with centriole, such as centrosomal protein of 135 kDa (CEP135), CEP152, CEP63, spindle and centriole-associated protein (SPICE), CP110, centrobin, CEP120, and CEP192 (Gonczy, 2012), are considered to govern the centriole assembly. The maintenance of a constant centriole number is critical for the progression of the cell cycle, and precisely controlled by numerous proteins which are involved in regulating centrosome duplication in the G1 and S phases, centrosome maturation in the G2/M phase and separation in the mitotic phase (Brownlee and Rogers, 2013). In human cells, PLK4, hsSAS-6 and STIL are three regulators necessary for centrosome duplication (Vulprecht et al., 2012). Following the activation of PLK4 and accumulation of STIL around the mother centriole, F-box protein FBXW5 stabilizes HsSAS-6 (Puklowski et al., 2011). In addition, several other proteins, such as CEP135, CPAP (Tang et al., 2009), γ-tubulin, CEP192, BRCA2, CP110 and its interaction protein USP33 (Li et al., 2013) that are essential for centrosome duplication, are recruited to the centriole, thus orchestrating centrosome duplication (Brownlee and Rogers, 2013). Additionally, cell cycle kinase CDK2 as well as potential partners of cyclin A and cyclin E are required for the two centrioles to split during centrosome duplication (Stearns, 2001). At the onset of mitosis, NEK2 and centrin are required for the sister centrosome disjunction as well as the formation of the two spindle poles during mitosis (Hinchcliffe and Sluder, 2001). Centrosome maturation is accompanied by the recruitment of many proteins to the centrioles and a dramatic expansion of the pericentriolar matrix (PCM) (Mennella et al., 2014). Phosphorylation is considered to be a key mechanism underlying centrosome maturation. The Polo-like kinases1 (PLK1) (Barr et al., 2004; Conduit et al., 2014) and Aurora kinases (Carmena and Earnshaw, 2003) have been identified as two important regulators of centrosome maturation. The specific phosphorylation of pericentrin (PCNT) by PLK1 results in the recruitment of many centrosomal proteins, such as γ-tubulin, Aurora A, PLK1, CEP192, and GCP-WD (γ-complex protein with WD repeats), to the centrosome during mitosis (Lee and Rhee, 2011). PCM is a matrix of proteins involved in centrosomal organization, microtubule nucleation and anchoring. The main components of PCM exist in the form of two proteins layers, one comprising a large number of coiled-coil proteins, such as pericentrin/pericentrin-like protein (PLP) and CEP152, with the other one including CEP215, γ-tubulin and CEP192 (Mennella et al., 2014). In PCM, γ-tubulin and other proteins such as γ tubulin complex protein (GCP) family can be assembled as γ-tubulin ring complexes (γ-TuRCs) for microtubule nucleation. The GCP family is also involved in the γ-TuRCs function, regulation and localization of γ-TuRCs (Kollman et al., 2011). The centrosomal protein pericentrin and the ninein-like protein (NLP) have been shown to anchor γ-TuRCs at the spindle poles (Zimmerman et al., 2004). Meanwhile, the precise components and regulators of γ-TuRCs remains incompletely understood.

Collectively, the identification of the structural and functional proteins of centrosome is clearly crucial for elucidating the structure of the centrosome and uncovering the underlying mechanisms in centrosome organization and regulation. Up to now, only a portion of the centrosome components have been detected (Tables S1, S2), and more efforts are required for the experimental validation of the remaining components. The centrosome in yeast cells is termed the spindle pole body (SPB), which is composed of a half-bridge for new SPB assembly, and three plaques, including an inner plaque for nuclear microtubules forms as the mitotic spindles originate, a central plaque spanning the nuclear membrane, and an outer plaque for cytoplasmic microtubules that used for karyogamy, nuclear positioning and spindle orientation (Seybold and Schiebel, 2013). The identified SPB proteins involved in the organization and regulation of SPB are listed in Table S2. However, there is still a large number of proteins located on the SPB that need to be further validated (Table 1).

The centrosome is a complex and precisely regulated organelle for bipolar spindle assembly, primary cilia formation, cell division and certain other cellular processes, including cell migration, protein degradation and axonal growth in human cells. More recent studies have shown that aberrant organization of centrosome resulting from defects in structural and functional proteins of the centrosome (Ganem et al., 2009; Nigg and Raff, 2009) is linked to neurodegenerative, Bardet–Biedl syndrome (Swaminathan, 2004), microcephaly (Marthiens et al., 2013), cystic kidney disease (Ong and Wheatley, 2003) and tumorigenesis (Marina and Saavedra, 2014). Thus, identification of the centrosomal proteins and clarification of the mechanisms underlying the centrosome assembly and regulation may lead to new drug targets, diagnostics or therapeutic approaches.

## The kinetochore

During mitosis in eukaryotic cells, a large number of proteins are assembled as a unique protein complex called the kinetochore, at the surface of the centromeric chromatin/centromere. The kinetochore functions as the binding site of the spindle microtubules to chromatin and directs sister chromatid segregation (Cheeseman and Desai, 2008). The protein components of the kinetochore modulate the connection between the centromeric chromatin and microtubules from the mitotic spindle to facilitate the proper segregation of the chromosomes during cell division (Gonen et al., 2012). According to the MiCroKiTS database and a comprehensive literature review (Cheeseman and Desai, 2008; Gonen et al., 2012), many kinetochore proteins have been identified in different species (Table 1). However, there are still a number of proteins localized at the kinetochore without any functional validation, as shown in the MiCroKiTS database.

The kinetochore is a complex and dynamic structure of variable size and shape. It is difficult to obtain the structural information on the complete kinetochore, so the structure is still not entirely clear. Previous studies have revealed that the overall positioning, main components and architecture of kinetochore are highly conserved from yeast to human (Quarmby and Parker, 2005). Many copies of centromeric proteins are assembled as a trilaminar kinetochore structure with the inner layer, a platform for kinetochore assembly that is located on the centromeric chromatin, the outer layer, responsible for the interaction with spindle microtubules, and the central layer, a region that links the inner and outer layers. In vertebrate cells, the inner layer consists of at least 18 centromeric proteins (Santaguida and Musacchio, 2009). Histone H3 variant centromeric protein A (CENP-A), also known as Cse4 in budding yeast, is one inner layer protein that may function as an early epigenetic marker for centromere localization and formation by making the centromeres distinct from the rest of the chromosome (Barnhart et al., 2011; Guse et al., 2011; Henikoff et al., 2014). CENP-A, together with CENP-B and CENP-C, are three main auto-antigens recognized by anticentromeric antibodies (Masumoto et al., 1989). Many other CENPs are also included in the inner layer, such as CENP-H, CENP-I, and CENP-K–W, all of which along with CENP-C colocalize with CENP-A and constitute the constitutive centromere-associated network (CCAN) (Cheeseman and Desai, 2008) (Table S3). Most of the components of the inner layer are evolutionarily conserved. They are responsible for keeping the kinetochore tethered to the centromere throughout the cell cycle and are essential for outer layer assembly (Carroll and Straight, 2006; Okada et al., 2006; Tanaka et al., 2009). The outer layer of the kinetochore is composed of several super-complexes, including Mis12, Ndc80 and Ska. The Mis12 complex provides the main platform for outer layer assembly, and consists of MIS12, NSL1, NNF1, and DSN1 (Screpanti et al., 2011). The Knl1 complex, which consists of KNL-1 and ZWINT, has been shown to recruit other outer layer proteins, such as spindle assembly checkpoint (SAC) proteins, CENP-F and the Rod–ZW10–Zwilch (RZZ) complex. The Ndc80 complex (NDC80, NUF2, SPC24, and SPC25) is one of the core binding sites of kinetochore-microtubules (kMTs) (Malvezzi et al., 2013). The Ska complex, composed of SKA1, SKA2, and SKA3, is essential for stabilizing kMT attachement (Welburn et al., 2009). In addition, the Knl1 complex, together with the Mis12 and Ndc80 complexes, forms the core of a highly conserved KMN network. This network is required for effective kMT attachment and force generation, and regulated by the Ska complex (the Dam/Dash complex in yeast) (Varma and Salmon, 2012). During mitosis, the components of the SAC, a mechanism that acts in response to unattached kinetochores, are recruited to the kinetochore monitor the correct kMT attachment by inhibiting the polyubiquitylation activities of the anaphase promoting complex (APC) (Peters, 2006). Several SAC components have been identified to date, including the non-kinase components Mad1, Mad2 and Bub3, the kinase components BubR1 (Mad3 in budding yeast), Bub1 and Mps1, the RZZ complex and other proteins (Lara-Gonzalez et al., 2012), as shown in Table S4. Among these components, Mad2 can interact with the APC activator of CDC20 to negatively regulate its function for the purpose of APC inhibition (Yu, 2002). In recent studies, several other mitotic protein kinases, including Aurora B (Chan et al., 2012) and PLK1 (Kang et al., 2006), PP2A phosphatase (Schmitz et al., 2010) and a number of nuclear pore proteins, including the Nup107–160 complex and SEH1 (D'angelo and Hetzer, 2008), have also been shown to transiently localize to the kinetochore during mitosis. They are involved in accurate segregation of chromosomes and controlling kinetochore function (Cheeseman and Desai, 2008), possibly by modulating checkpoint signaling. As the main structural features of kinetochores are conserved from yeast to human, the kinetochore also consists of the inner and outer layers in yeast, and the kMT attachment is regulated by numerous SAC proteins (Tables S3, S4). Among the components of the kinetochore found in yeast and human, the Ndc80 complex and some of the SAC proteins are highly conserved and exist in both species, indicating the importance of these proteins for correct chromosome segregation during cell division.

A combination of biochemical, fluorescence-microscopy and electron microscopy (EM) studies has led to the proposal of several structural models of the kinetochore with only weak supporting evidence. However, in recent studies, the first three-dimensional images of the kinetochore core structure have been obtained from budding yeast. These images show that the size of the kinetochore is approximately 126 nm, with a large central hub surrounded by multiple outer globular domains that form a ring-like structure around the microtubules (Gonen et al., 2012). This finding is important and extends the knowledge of the kinetochore. To further the understanding of the assembly process of the kinetochore and the mechanisms underlying chromosome segregation, additional kinetochore components and higher resolution images of kinetochore are needed to assist the elucidation of the structure and regulatory network. These are key elements in advancing our understanding of the mechanisms of the kinetochore-associated diseases, such as cancer, and may contribute to the development of early-stage clinical treatments (Gonen et al., 2012).

## The midbody

During cytokinesis, many proteins promote furrow ingression, dividing one cell into two daughter cells still connected by midbody, a cellular substructure contains many transient protein complexes formed at the narrow intracellular bridge (Steigemann and Gerlich, 2009). The midbody is generally considered to be an important structure for directing the abscission and completely separating the two daughter cells at the final stage of cytokinesis (Pohl and Jentsch, 2008). However, more functions of the midbody are still unclear. According to recent studies, the midbody may also be involved in cell-fate determination. Morphologically, the midbody is a dense structure formed by a tightly packed anti-parallel microtubule array, and many proteins are recruited to this site to assist in the cytokinesis process (Mullins and Biesele, 1977; Steigemann and Gerlich, 2009). However, the current knowledge of the midbody components and the way the midbody proteins are organized is limited. To further clarify the functions and the processes of assembly and regulation of the midbody, the primary task is to identify its protein components. Although there are approximately 229 proteins identified as being associated with the midbody in human cells, and 133 proteins in yeast cells (Table 1), there are still many remaining components that urgently need to be uncovered and validated.

Previous studies have shown that the midbody proteins are organized in three parts, the bulge, the dark zone and the flanking zone (Mullins and Biesele, 1977; Steigemann and Gerlich, 2009). The bulge is at the center of the midbody, containing few bundled anti-parallel microtubules and various proteins. In human cells, centralspindlin, a key component of the bulge, is a complex of the human GTPase-activating protein MgcRacGAP and Mitotic kinesin-like protein 1 (MKLP1). Centralspindlin is essential for the midbody formation and links the midbody to the plasma membrane. Many of the identified bulge proteins are associated with centralspindlin. The ADP-ribosylation factor 6 (ARF6) GTPase can interact with centralspindlin and may be respectively responsible for midbody stabilization (Joseph et al., 2012). The Rho guanine nucleotide exchange factor (RhoGEF) Ect2 is also a centralspindlin-interacting protein and localizes at the bulge to facilitate midbody abscission (Yuce et al., 2005). The coiled-coil protein centriolin, recruited to the midbody by centralspindlin, is important for integrating the process of membrane-vesicle fusion with abscission by interacting with the exocyst components and SNARE complexes (Gromley et al., 2005). Another centralspindlin binding protein is a centrosomal protein of 55 kDa (CEP55) that is persistently localized at the midbody bulge during cytokinesis. The tumor-susceptibility gene 101 (TSG101) has been observed at the bulge. TSG101 and another midbody protein called Alg2-interacting protein X (ALIX) are associated with CEP55, and are proposed to be responsible for recruiting ESCRT-III components to the dark zone and thus assisting with the midbody abscission (Morita et al., 2007; Lee et al., 2008; Elia et al., 2011). The dark zone is a narrow region in the center of the midbody where antiparallel microtubules overlap. The microtubule-associated protein regulator of cytokinesis 1 (PRC1), in association with a microtubule-based motor protein of kinesin superfamily protein member 4 (KIF4), colocalizes at the midbody dark zone and together they are essential for cytokinesis (Kurasawa et al., 2004). Wnt5a signaling is important for stabilization. In recent studies, Wnt receptor Frizzled 2 (FZD2), which has been observed in the dark zone and has a similar localization pattern as the ESCRT-III subunit of CHMP4B, may regulate ESCRT-III localization via a Wnt5a-mediated β-catenin-independent signaling pathway (Fumoto et al., 2012). The midbody flanking zone resides outside of the dark zone, containing multi-proteins (Hu et al., 2012), such as the negative cytokinesis-regulator of centromere protein E (CENPE) (Liu et al., 2006), mitotic kinesin-like protein 2 (MKLP2) that regulates the localization of the chromosomal passenger complex (CPC) during cytokinesis (Gruneberg et al., 2004), and a CPC subunit of the Aurora B kinase-mediated abscission checkpoint (Steigemann et al., 2009). In yeast cells, the bud neck, which is analogous to the midbody, is responsible for cytokinesis and abscission (Guertin et al., 2002). And the main components of the organism are evolutionarily conserved from yeast to vertebrate (Otegui et al., 2005).

Actually, many components of each substructure of the midbody and bud neck subregions listed in Tables S5, S6 display a dynamic localization pattern, but the detailed composition of midbody and bud neck is still not known. In human cells, the midbody contains secretory or membrane-trafficking proteins, actin-associated proteins, microtubule-associated proteins, kinases proteins, and other uncharacterized or other function proteins, involved in many processes, such as the cytoskeleton, lipid rafts and vesicle trafficking (Skop et al., 2004). In addition, recent studies indicate that the functions of the midbody are not only related to abscission, but also involved in patterning, morphogenesis and development during embryogenesis (Chai et al., 2012). The accumulation of midbodies has been shown to correlate with the pluripotency of stem cells and to increase the tumorigenicity of cancer cells, while in differentiated cells, the midbody is degraded through an autophagy pathway (Ettinger et al., 2011; Kuo et al., 2011; Schink and Stenmark, 2011). Thus, identification of the midbody components is essential for advancing our knowledge of midbody and cell-fate determination, and also for exploring new therapeutic strategies for midbody related diseases treatment, such as cancer.

## Discussion

**Figure 1:**
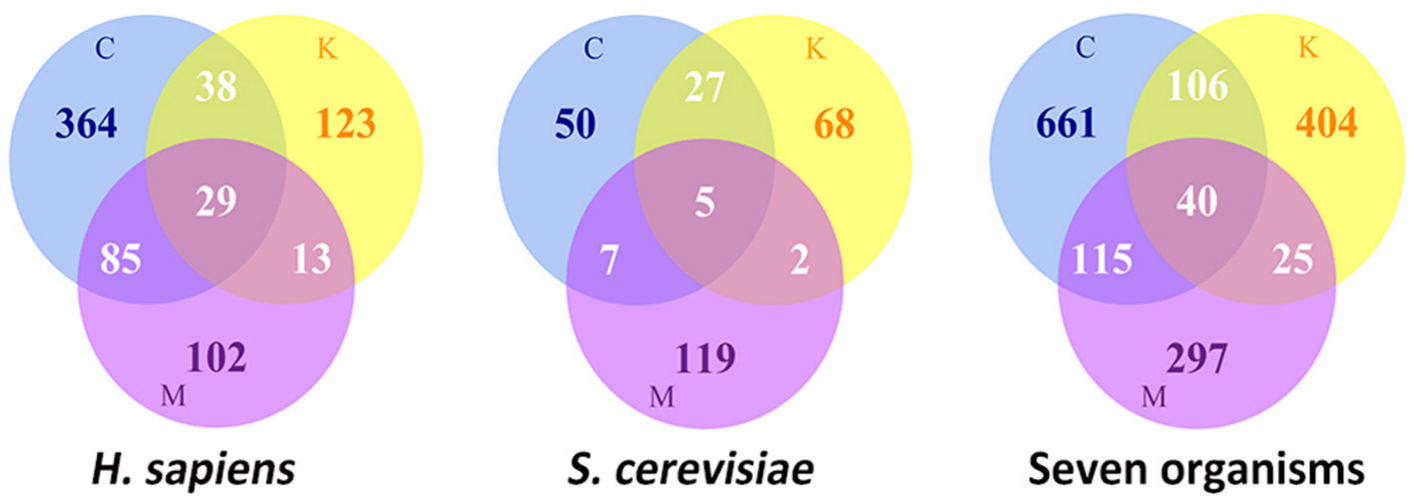
**The statistics of Location distributions of MicroKiTS proteins**. C refers to Centrosome. K refers to Kinetochore. M refers to Midbody. Seven organisms include of *H. sapiens*, *S. cerevisiae*, *C. elegans*, *D. melanogaster*, *X. laevis*, *M. musculus*, and *S. prombe*.

A large number of proteins have been shown to participate in the process of cell division and spatiotemporally assemble as super-complexes at defined subcellular localizations, such as kinetochores at the centromeric chromatin, the centrosome near the nucleus, and the midbody between two daughter cells. According to the MiCroKiTS database search, during cell division, there are a total of approximately 754 identified proteins localized at the organelles of the centrosome, kinetochore and midbody in *Homo sapiens*, and 278 in *Saccharomyces cerevisiae* (Figure 1). Despite the fact that the protein components of each organelle are recruited to a specific subcellular localization, some proteins exhibit multi-localization in various species. Collectively, there are approximately 165 proteins which have more than one subcellular localizations in *Homo sapiens*, while there are 41 proteins in *S. cerevisiae*.

Proteins with multiple localizations are the key factors for mediating the communication between the organelles. In human cells, Ndc80 complex dynamically localizes at centrosome, and then concentrates at centromere and becomes a stable component of kinetochore until completion of the mitosis (Hori et al., 2003). Ndc80 complex is required for the stable kinetochore-spindle microtubule attachments, which controls the chromosome alignment and segregation in mitosis (Wei et al., 2005). The kinetochore protein components of INCENP (Cooke et al., 1987), CENP-A(Liu et al., 2013) and Aurora B (Kimura and Okano, 2005) for the chromosome biorientation, and the centrosome proteins of BARD1 (Ryser et al., 2009), BRCA2 (Daniels et al., 2004) and CEP55 (Fabbro et al., 2005), can be recruited to the midbody for the progression of cytokinesis. PLK1 (Cdc5 in yeast), a key mitotic regulator that phosphorylates substrate proteins on several different mitotic structures in human cells, first localizes at the centrosome before associating with kinetochore, and then is recruited to the midbody (Petronczki et al., 2008). The dynamic localization of PLK1, mediated by the polo-box domain (PBD) and kinase activity, is critical for chromosome alignment, spindle assembly and cytokinesis (Petronczki et al., 2008; Liu et al., 2012). A ubiquitin-ligase complex of APC and the HECT E3 ligase Smurf2, both of which control the progression of mitosis and cytokinesis through ubiquitin modification of substrate proteins and thus altering the protein localization and stability, have also been found to be dynamically localized at the centrosome, kinetochore and midbody (Kurasawa and Todokoro, 1999; Osmundson et al., 2008). In yeast cells, 5 proteins, including Cdc5 (Snead et al., 2007), protein phosphatase 2A regulatory subunit RTS1 (Gentry and Hallberg, 2002) and TPD3 (Gentry and Hallberg, 2002), Casein kinase I homolog HRR25 (Lusk et al., 2007) and protein phosphatase PP1-2 (Bloecher and Tatchell, 2000), are spatiotemporally recruited to the SPB, kinetochore and bud neck, and precisely regulate the cell division progression by altering the phosphorylation state of the substrates proteins. The subcellular localization determines the biological activities of multi-localized proteins through controlling the access of these proteins to different interaction partners, and is critical for the formation of the dynamic protein-protein interaction network to govern the process of the cell division/mitosis. Meanwhile, the posttranslational modifications (PTMs), including phosphorylation and ubiquitylation, as well as altering of the subcellular localizations, are essential mechanisms used by multi-localized proteins to diversify function and regulate cell division. A latest analysis of the dynamics of proteome and phosphoproteome during the cell division of the fission yeast revealed that changes of proteome level are weak, whereas changes of protein phosphorylation states are the predominant events occurred in mitosis, indicating that phosphorylation is probably associated with the functions and localizations of the proteins, which are involved in regulating mitotic progression and completion(Carpy et al., 2014). Additionally, the progresses in proteome-wide analysis of ubiquitination modifications in cell division demonstrated that ubiquitination, which affect protein stability, activity, and localization, plays an important role in regulating the mitotic progression (Chuang et al., 2010; Merbl et al., 2013). Certainly, the current understanding of the mechanisms used by multi-localized proteins to dynamically control the formation and functions of subcellular structures is still limited. Future studies are needed to identify the components of the subcellular structures as well as the multi-localized proteins, and also to characterize their functions, on–off mechanisms and crosstalk.

## Author's contributions

Yueyuan zheng, Junjie Guo, and Xu Li contribute to the literature collection and help to draft the manuscript. Mingming Hou, Yubin Xie, Xuyang Fu, Shengkun Dai, and Rucheng Diao participate in drafting the manuscript and revising it critically for important intellectual content. Yanyan Miao writes the manscript and interprets the data. Jian Ren contributes to the conception and design of the work.

### Conflict of interest statement

The authors declare that the research was conducted in the absence of any commercial or financial relationships that could be construed as a potential conflict of interest.
