# Supplementary material for: An integrated overview of spatiotemporal organization and regulation in mitosis in terms of the proteins in the functional supercomplexes
Source: Front Microbiol. 2014 Oct 29;5:573. doi: 10.3389/fmicb.2014.00573 (PMC4212687; doi:10.3389/fmicb.2014.00573)
Supplement: Supplementary file 1 [file Table1.DOC]

Table S1 - The core and related Centrosomal proteins in human*.

| **Centrosomal protein** | |
| --- | --- |
| Scaffolding component of the centrioles | HsSAS6 (21277013); CEP135 (23199753); STIL (22892665) |
|  | Related proteins required for assembly: POC1B (23015594); POC1A (23015594) |
| Distal appendages | CEP164 (23348840); ODF2 (12510196); CEP89 (23348840); CEP83 (23348840); SCLT1 (24295827); FBF1 (23348840, 24295827) |
| Sub-distal appendages | ODF2 (12510196); ninein (12510196); TUBE1 (12510196); CEP170 (17954613); centriolin (12732615); CC2D2A (24947469) |
| Pericentriolar material  (PCM) | CPAP (24268653), pericentrin/PLP (24268653), Cep152 (24268653), Cep215 (24268653), Cep192 (24268653), γ-tubulin ring complex (TUBG1, TUBG2, GCP2, GCP3, GCP4, GCP5, GCP6) (24268653) |
|  | Related proteins required for PCM: Kiz (16980960); SSX2IP (23816619) |
| **Central Process** | |
| Centrosome duplication | Proteins involved in duplication: HsSAS6 (22892665); CPAP (22892665); CEP135 (22892665); CEP152 (23936128); CEP63 (23936128); CP110 (17719545); CEP97 (17719545); centrobin (21576394); CEP120 (17015463); CEP192 (22691849); USP33 (23486064); FBXW5 (21725316); ROCK2 (17015463); NPM (17015463); CETN2 (12176356); PCM1 (12403812); TUBE1 (12510196) |
|  | Regulatory proteins: PLK4 (16244668); SPICE (20736305); STIL (22349705); RAB6C (20064528); BRCA2 (17286961); CDK2 (19238148); cyclin-A/E (11371338); fibrocystin (20554582); CEP76 (19460342); FBX1 (20596027); PLK2 (22399798); LATS2 (15343267); Nek2 (11358861); centrin (11358861) |
| Centrosome maturation | Regulatory proteins: TNKS1 (22864114); Aurora A (24268653); PLK1 (24268653); Cep192 (24268653); CDK5RAP2 (24268653); SSX2IP (23816619) |
| Cilium assembly | Proteins involved in assembly: POC1A; POC1B; IFT20 (16775004); IFT81 (23990561); CEP41 (22246503); CEP83 (23348840); CEP89 (23348840); FAM161A (22940612); FOPNL (20551181); CEP162 (23644468); CEP164 (17954613) |
| Microtubule nucleation | Proteins involved in microtubule nucleation: γ-tubulin ring complex (TUBG1, TUBG2, GCP2, GCP3, GCP4, GCP5, GCP6); NEK7 (17586473); CEP72 (19536135); NEDD1 (19029337); CPAP (15047868); CKAP5 (12569123); RABGAP1 (10202141); NINL (12852856) |

* All proteins are from published literatures and MicroKiTS database searches. By parsing from the UniProt database and Gene Ontology (GO) terms, the proteins are classified into different categories according to their Cellular Component and Biological Process annotations. The PubMed IDs of related literatures are listed in the brackets.

Table S2 - The core and related Centrosomal proteins in budding yeast*.

| **Spindle pole body (SPB) protein** | | | |
| --- | --- | --- | --- |
| Inner plaque of SPB | γ-tubulin complex (TUB4, SPC97, SPC98) (9670012); SPC110 (9215630) | |  |
| Central plaque of SPB | BBP1 (10654940), SPC29 (10654940); NBP1 (16436507); SPC110 (9215630); SPC42 (9215630); calmodulin (10339566) | |  |
| Outer plaque of SPB | CNM67 (9571234); SPC72 (9670012); γ-tubulin complex (TUB4, SPC97, SPC98) (9215630) | |  |
| Membrane Components and the Half-Bridge | KAR1 (8070654); CDC31 (8070654); MPS3 (12486115); SFI1 (10455233); MPS2 (16436507); NDC1 (16436507) | |  |
| Cytoplasmic microtubules | BIM1 (10352017); PAC1 (12566428); CIK1 (9153752); KAR3 (2138512); DYN3 (14764870); TUB1 (9488492); TUB3 (3540600); TUB2 (9488492); TUB4 (9215630); PAC11 (15642746); KIP3 (9693366); CDC11 (18826657); NDL1 (15965467); KIP2 (9693366); DYN1 (12566428); DYN2 (21633107); | |  |
|  | Related proteins involved in cytoplasmic microtubules: KAR9 (9442113); SPC72 (17967947) | |  |
| Nuclear microtubules | KIP3 (9693366); SLK19 (10835382); TUB4 (9215630) | |  |
| **Central Process** | |  |  |
| Microtubule organizing center | γ-tubulin complex(TUB4, SPC72, SPC97, SPC98)(9670012); CIK1(10087265); DAD1(15664196); KIP3(10525539); SLK19(10427094); STU2(9606209) | |  |
| SPB duplication | Proteins involved in duplication: SPC110, SPC42, calmodulin; BBP1; CDC31; KAR1; SPC29, MPS1 (19269975); MPS2; MPS3; NDC1 (15075274); SFI1 | |  |
|  |  |  |  |
| Microtubule nucleation | Proteins involved in microtubule nucleation: CDC31; CNM67; SPC105, SPC110, SPC24, SPC29, SPC98, SPC97, SPC42, SPC25, STU1, STU2, NUF2 (9153752); SPC72 (18573877); TBG1 | |  |

* All proteins are from published literatures and MicroKiTS database searches. By parsing from the UniProt database and Gene Ontology(GO) terms, the proteins are classified into different categories according to their Cellular Component and Biological Process annotations. The PubMed IDs of related literatures are listed in the brackets.

Table S3- The core and related centromere/kinetochore proteins in human*.

| Central Component | Proteins |
| --- | --- |
| Inner/Central kinetochore | CENPA (21768289); CENPB (2808515); CCAN(CENPC, CENPH, CENPI, CENPK, CENPL, CENPM, CENPN, CENPO, CENPP, CENPQ, CENPR, CENPS, CENPT, CENPU, CENPV, CENPW) (18097444) |
|  | Related proteins required for assembly : MIS18 complex (MIS18BP1; MIS18B; MS18A) (17199038) |
| Outer kinetochore | Knl1 Complex (KNL1, ZWINT) (21353556); Mis12 Complex (DSN1, MIS12, NSL1, PMF1) (21353556); Ndc80 Complex (NDC80, NUF2, SPC24, SPC25) (23334295); Ska1 complex (SKA1, SKA2, SKA3) (19289083) |
|  | Related proteins required for assembly: CENPK (18045986); Sgt1 (15133482) |
| Spindle assembly checkpoint (SAC) | BUBR1 (23174302); BUB3 (23174302); CDC20 (23174302); BUB1 (23174302); MAD1 (23174302); MAD2 (23174302); MPS1 (23174302); RZZ complex (KNTC1/ROD, ZW10, ZWILCH) (12686595); PICH (17218258) |
|  | Related regulatory proteins: Aurora B (22371557); INCENP (11453556); PLK1 (17081991)  Related proteins required for SAC: NDC80 complex (NDC80, NUF2, SPC24, SPC25); ZWINT; KNL1; NEK2 (14978040); SPDL1 (17576797) |
| Kinetochore-microtubule attachment (kMT) | Ska1 complex (SKA1, SKA2, SKA3 )(19289083) |
|  | Related regulatory proteins: BUB3 (18199686); NEK2; SKAP (19667759); SPAG5 (21402792); B56-PP2A (21874008)  Related proteins required for K-MT: KNL1; NUP43 (17363900); NUP37 (17363900); SEH1 (17363900); CHAMP1 (21063390) |

* All proteins are from published literatures and MicroKiTS database searches. By parsing from the UniProt database and Gene Ontology (GO) terms, the core proteins are classified into different categories according to their Cellular Component. The PubMed IDs of related literatures are listed in the brackets.

Table S4 - The core and related centromere/kinetochore proteins in budding yeast*.

| Central Component | Proteins |
| --- | --- |
| Inner kinetochore | CSE4 (10958698); CBF3 complex (CEP3, CTF13, CBF2, SKP1) (15090617); MIF2 (7579695) |
| Central kinetochore | COMA complex (AME1, CHL4, CTF19, CTF3, IML3, MCM16, MCM21, MCM22, NKP1, NKP2, OKP1) (14633972) |
| Outer kinetochore | SPC105 complex (KRE28, SPC105) (14565975);  MIND complex (DSN1, MTW1, NNF1, NSL1) (14633972);  NDC80 complex (NDC80, NUF2, SPC24, SPC25) (14633972);  DAM1/DASH complex (ASK1, DAD1, DAD2, DAD3, DAD4, DAM1, DUO1, HSK3, SPC19, SPC34) (11756468) |
| Spindle assembly checkpoint (SAC) | MAD3 (10704439); BUB3 (10704439); CDC20 (10704439); MAD2 (10704439); MAD1 (10837255); BUB1 (10837255); SGO1 (15637284); SPC105 complex (KRE28, SPC105 )(19893618) |
|  | Related proteins required for SAC : MIND complex(DSN1, MTW1, NNF1, NSL1); NDC80 complex(NDC80, NUF2, SPC24, SPC25) |
| Kinetochore-microtubule attachment (kMT) | DAM1/DASH complex (ASK1, DAD1, DAD2, DAD3, DAD4, DAM1, DUO1, HSK3, SPC19, SPC34) (19893618) |
|  | Related regulatory proteins: AME1; CTF19 (10189365); MPS1 (11278681)  Related proteins required for K-MT: DSN1 (14633972); IPL1 (12408861); SLI15 (11853667); OKP1 |

* All proteins are from published literatures and MicroKiTS database searches. By parsing from the UniProt database and Gene Ontology (GO) terms, the core proteins are classified into different categories according to their Cellular Component. The PubMed IDs of related literatures are listed in the brackets.

Table S5 - The core Midbody proteins in human*.

| **Midbody protein** | |
| --- | --- |
| Bulge zone | Centralspindlin complex (MKLP1, MgcRacGAP1, CEP55, ARF6) (22580824,19962307); TSG101 (18367816); NIR2 (12077336, 15125835); AMSH (18388320); ECT2 (20348415,16803869); BRUCE (18329369) |
| Dark zone | PRC1 (12082078); KIF4 (22278743); Supervillin (20309963); ALIX (22771033,17556548); Syne-1 (14709720); Rab6-KIFL (11060022); PLK1 (22278743); CHK2 (12493754); hFZD2 (22825874); CHMP4B (22825874) |
| Flanking zone | MKLP2 (23562843); Aurora B (23562843,24857377); Syntaxin16 (19887622); EVI5 (17099728); DMA1 (18171988); h-warts (10518011); INCENP (24937015,15796717); Survivin (15477601); hSGT (14729056) |
| **Central Process** | |
| Midbody formation | Centralspindlin complex (MgcRacGAP, MKLP1) (23235882); ARF6 (22580824); CHO1 (12058052); Annexin 11 (15197175); Ect2 (16803869); Filamin A, BRCA2 (22771033); PRC1 (16603632); CEP55 (16790497); Rab6-KIFL (11060022); KIF4 (15297875); Survivin (15477601) |
|  | related regulatory proteins: Aurora B (15854913); INCENP (15796717); MKLP2 (12939256); PLK1 (22621898) |
| Abscission | TSG101/ESCRT-I and ALIX (17853893);  ESCRT-0 complex (STAM1, STAM2, HSG) (21940792);  ESCRT-I complex (TSG101, VPS28, VPS37a-d, MVB12A) (21940792);  ESCRT-II complex (SNF8, VPS25, VPS36) (21940792);  ESCRT-III complex (CHMP2A, HMP4A, CHMP5)(21940792);  VPS4 (24814515); Centralspindlin complex (18511905); Centriolin (16213214); SNARE components (syntaxin-2, VAMP8) (12737809); Syntaxin 16 (24109596); FIP3 (18511905, 21790911); Ect2 (18511905, 16803869); BRUCE (18329369); SEPT9 (21059847) |
|  | related regulatory proteins:CEP55(16790497); BRCA2 (22771033); AMSH (18388320, 21827950); UBPY(18388320); Mob1 (22454515); MNK1 (22454512); ARF6/Rab35 GTPase (22226746); ANCHR, Aurora-B (24814515); spastin (21310966); PLK1 (21079244); MKLP2 ( 22771033); Wnt5a signaling (hFZD2, Dvl2) (22825874 ) |

* All proteins are from published literatures and MicroKiTS database searches. By parsing from the UniProt database and Gene Ontology (GO) terms, the core proteins are classified into different categories according to their Cellular Component and Biological Process annotations. The PubMed IDs of related literatures are listed in the brackets.

Table S6 - The core Midbody proteins in budding yeast*.

| **Bud neck protein** | | |
| --- | --- | --- |
| Daughter site of the bud neck | Septins (11390675); RGA1 (17347519); AXL2 ( 8846915); BUD9 (11514631); RAX2 (11110666); HSL1 (10805747); HSL7(10805747); SWE1 (10805747); DBF2 (10984431); MOB1 (11434459); RVS161 (9566960); KCC4 (11165249); CDC5 (10594031); BNI4 (20197406); CHS2 (16847101); BUD8 (11378394); BUD5 (11313501) | |
| Mother-bud neck | BNI5 (12215547); CHS2 (16847101); CHS3 (8909536); CYK3 (10959846); BUD3 (7730410); MYO1 (9732290); IQG1 (9382845); RAX2 (11110666); CLB2 (11171327); YCK2 (10198058); Septins (11390675); GIN4 (9813093); ELM1 (10234786); SMT3 (10579719); BNI4 (9314530); CHS4 (9314530); MLC1 (11082046); HOF1 (10679017); BUD4 (8707826); BUD2 (10444589); BUD6 (9247651); DBF2 (10984431); RVS161 (9566960); BNR1 (20147448); SYP1 (11014808); GLC7 (10747092); CDC5 (10594031); BUD5 (11378394) | |
| **Central Process** | |  |
| Midbody formation | Buk proteins: AXL1, AXL2, BUD3, BUD4, BUD8, BUD9, RSR1/BUD1, BUD2, BUD5 (15380095); YCK2 (10198058); Septins: CDC12, CDC11, CDC10, CDC3, SHS1 (12058072); Myosin ring assembly: MYO1, MLC1, MLC2, ACT1, PFY1, BNI1, BNR1, COF1, LQG1, HOF1, RHO1(15380095); Chitin synthesis: CHS2, BNI4, CHS4, CHS3 (9990311, 12529424, 9314530, 8909536); | |
|  | Related regulatory proteins: Gin4, Elm1, Cla4, Hsl1, Nap1, Cdc42, Hsl7 (12058072) | |
| Abscission | ESCRT-0 complex (HSE1, VPS27) (19325624);  ESCRT-I complex (STP22, VPS28, SRN2, MVB12) (15086794, 22240455); VPS4 (22240455);  ESCRT-II complex (VPS22, VPS25, VPS36) (15086794, 22240455);  ESCRT-III complex (VPS2, VPS20, VPS24, SNF7) (15086794, 22240455); | |
|  | Related regulatory proteins: RHO1 (23878277); IPL1, BOI1, BOI2 (16615892) | |

* All proteins are from published literatures and MicroKiTS database searches. By parsing from the UniProt database and Gene Ontology (GO) terms, the core proteins are classified into different categories according to their Cellular Component and Biological Process annotations. The PubMed IDs of related literatures are listed in the brackets.
